# Global pelagic dispersal traits datasets for early-life stages of marine fishes

**DOI:** 10.1038/s41597-026-07768-1

**Published:** 2026-09-08

**Authors:** Eliot Ruiz, Marion Thémèze-Leroy, Franck Ferraton, Jacques Panfili, Jean-Dominique Durand, Michel Kulbicki, Juliette Silhol, Austin Bernard, Raphaël Thomas, Théo Morisson, Yannis Djouldem, Elvin Baptiste, Pierre-Yves Pascal, Lucie Vanalderweireldt, Sébastien Cordonnier, Amélia Chatagnon, Pierre-Louis Rault, Prune Chareyre, Leila Leon, Iris Leone, Ylana Fouchan, Stéphanie Manel, Laureline Boulanger, Benjamin Victor, Séverine Albouy-Boyer, Thomas Le Berre, Charlotte R. Dromard, Loïc Pellissier, Camille Albouy, Fabien Leprieur

**Affiliations:** 1 MARBEC, Univ Montpellier, IRD, IFREMER, CNRS, Montpellier, France; 2 ENTROPIE, IRD, University of Reunion, CNRS, IFREMER, University of New Caledonia, Noumea, New Caledonia; 3 Institute of Systematic, Evolution and Biodiversity, National Museum of Natural History, University of the Antilles, Pointe-à-Pitre, Guadeloupe, France; 4 Ecosystem and Landscape Evolution, Institute of Terrestrial Ecosystems, Department of Environmental Systems Science, ETH Zürich, Zürich, Switzerland; 5 Unit of Land Change Science, Swiss Federal Research Institute WSL, Birmensdorf, Switzerland; 6 UMR BOREA, CNRS 7208, MNHN, UPMC, UCBN, IRD 207, University of the Antilles, Pointe-à-Pitre, Guadeloupe, France; 7 CEFE, University of Montpellier, CNRS, EPHE-PSL University, IRD, Montpellier, France; 8 Institut Universitaire de France, Paris, France; 9 Ocean Science Foundation, Irvine, CA 92604 USA; 10 Guy Harvey Research Institute, Nova Southeastern University, Dania Beach, FL 33004 USA; 11 Freelance Marine Consulting, Wallisellen, Switzerland; 12 Reefscapers Marine Consulting society, Malé, Maldives

## Abstract

Although larval stages ensure most connectivity between demersal fish populations, dispersal traits are increasingly less measured, despite the importance of correctly parametrizing biophysical models for spatial conservation planning. Moreover, existing data are scattered across a vast multidisciplinary literature. To address this mismatch between need and availability, we compiled data from 3,201 references into multiple datasets covering 35 key pelagic dispersal traits across fish early-life stages. We specifically compiled data on swimming speed, vertical position, and delayed settlement in demersal fishes (rafting or pelagic juveniles), as these traits exert a major yet understudied influence on dispersal scale. We systematically reported all descriptive statistics, enabling robust fitting of probability density functions to introduce particle-level variability in Lagrangian models. Overall, datasets comprise 32,988 species-level (1,709 for higher-taxon-level) records for 6,841 marine fish species worldwide, including 1,155 unpublished records (other teams), and 313 records newly collected during two fieldworks (Maldives & Guadeloupe). We also digitized 12,322 individual-level age-at-length/weight records from rearing experiments, enabling temperature-dependent growth model fitting to forecast global warming impacts on dispersal.

## Background & Summary

Over the past 15 years, the study of marine connectivity has rapidly emerged as a key field of research in marine biology and oceanography^1,2^, particularly in the context of global change^3–5^. Increasing evidence indicates that past flows of genes, energy, and biomass among populations under the influence of both abiotic and biotic factors have shaped current biogeographic patterns^6^. Similarly, future fluxes will critically influence species resilience^7,8^, ultimately impacting fundamental ecosystem services on which our societies depend^9,10^. Connectivity among populations is of particular importance for marine organisms with a bipartite life cycle (*i.e*., dispersing as meroplankton before settling in coastal habitats) such as the vast majority of demersal marine fishes^11^. Indeed, most organism dispersal occurs during the early pelagic phase of demersal fishes (up to >1000 km^12,13^) whereas adult movements are more limited (often < 1 km^14^). This observation justifies the need for interconnected Marine Protected Area (MPA) within a network to maintain gene flow among distant populations^15^, while efficiently replenishing unprotected surroundings^16,17^.

To better estimate population connectivity, Lagrangian models offer a powerful and cost-effective tool to simulate organism movement driven by ocean currents^18^. These models simulate the dispersal of millions of particles under the influence of ocean currents and biological species traits. Among the few model validations against genetic data^19^, the only one that accounted for active larval behaviors demonstrated that realistic outcomes are achieved only when these behaviors are included and accurately parameterized^20^. The key behaviors to consider are vertical positioning of each life stage, which reduces current influence at depth^21^, and horizontal dispersal capacity, driven by swimming speed, endurance, and orientation precision^22,23^, but these parameters are all difficult or costly to measure^24,25^. Generally, limited knowledge of early life pelagic stages (including all stages from embryo to juveniles) hinders model application to most species, since only ~35% of marine fish larvae are described^26,27^. Indeed, fish larvae are difficult to capture (*e.g*., patchiness, net avoidance, sparse offshore/deep distribution), to identify morphologically (*e.g*., unreliable meristic counts, major ontogenetic changes), and to rear in captivity. Fewer than 3% of marine fish species were cultured to metamorphosis^28^, and just a small fraction of them are commonly raised, explaining why 93% of traded marine fishes are wild-caught^29^, while they are also heavily overfished for food^30,31^. Overall, existing data on life history traits of fish larvae are fragmented across disciplines, often outdated, or affected by evolving species names, making them very difficult to find, access, integrate and use. Therefore, it was not only essential to aggregate all scattered information about fish larvae in a single dataset, but also to measure new ones during two fieldworks in Guadeloupe (Caribbean Sea) and the Maldives (Indian Ocean), by prioritizing species with missing data.

Previous attempts to summarize various early life stage traits relevant for dispersal modeling (referred to here as “dispersal traits”) have mainly consisted of identification books (*e.g*.^26,32^) and aquaculture reviews within books (*e.g*.^33,34^) or articles (*e.g*.^35,36^). Most other major reviews focused on a single trait type (*e.g*.^37–39^) or provided reference lists without the underlying data (*e.g*.^40,41^). Remaining sources, mainly articles, of dispersal traits generally focus on a single species, with relevant information dispersed throughout the text, significantly increasing the time required for data extraction and the chances of missing some traits. Age-related data are generally obtained from rearing experiments, except for the Pelagic Larval Duration (PLD) typically inferred from otoliths (*i.e*., calcified inner ear structures involved in balance), which defines the timing of the concurrent morphological and ecological metamorphosis during settlement in many demersal fishes. However, the term PLD becomes ambiguous for several families that undergo metamorphosis after settlement (*e.g*., Haemulidae, Carapidae, Labridae & Pleuronectiformes^42–45^). It is also ambiguous for species that may remain in the pelagic environment during their juvenile stage (*e.g*., Holocentridae, Mugilidae, Mullidae, Diodontiformes^11^), or even as adults if they settle on a flotsam or a pelagic animal^46^. Additionally, PLD excludes by definition the egg phase, which also contributes to dispersion in the predominant case of pelagic spawning^47^.

**Fig. 1:**
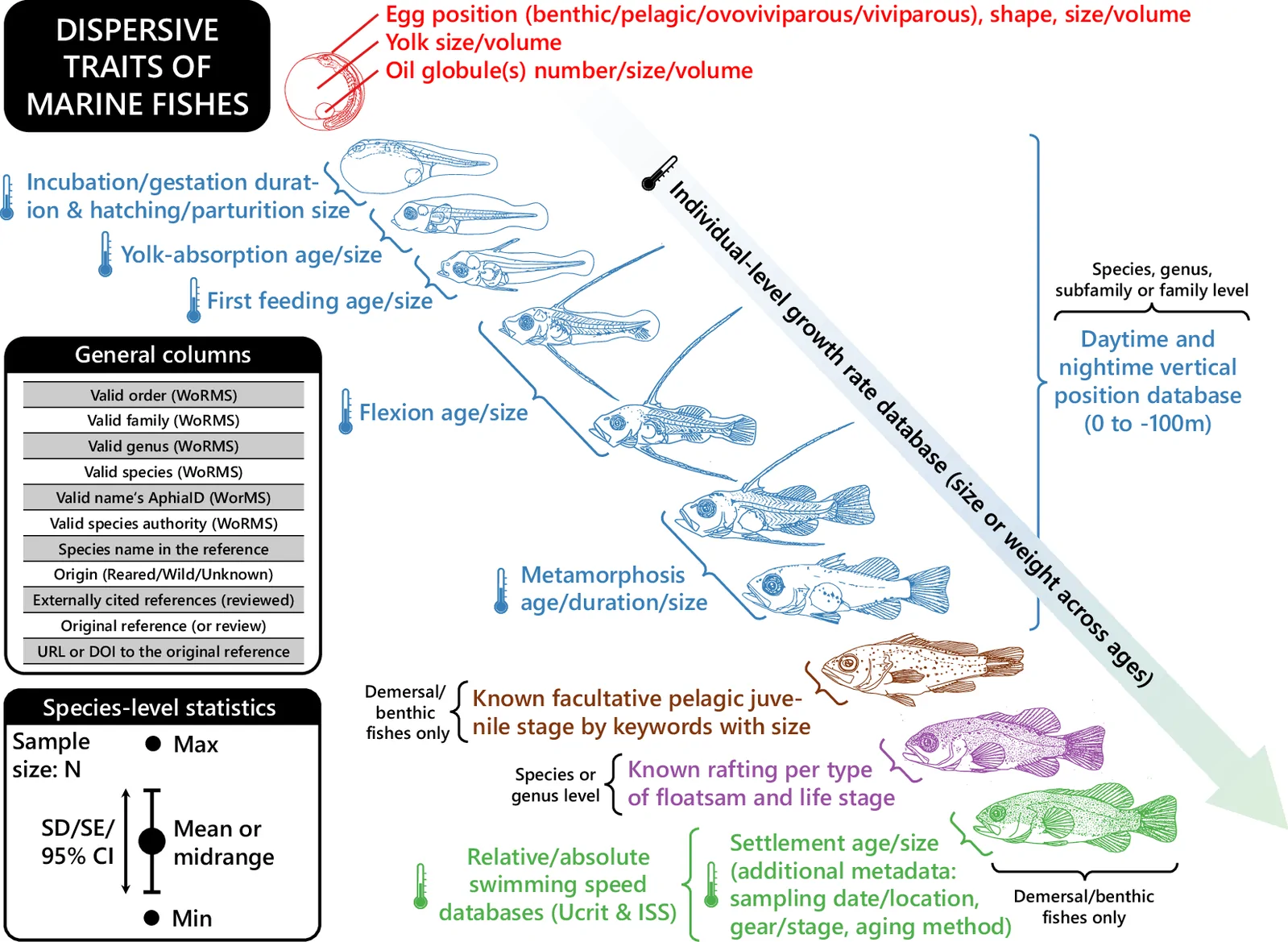
New dispersal traits datasets across ontogeny, from the egg stage (red), to the end of the larval stage (blue) and the end of the pelagic stage (green) in the case of demersal/benthic marine fishes. Settlement can indeed occur before metamorphosis, or after, due to facultative pelagic life prolongations such as the pelagic juvenile phase (brown) or the possibility to settle on rafts (purple). Except for the growth rate dataset, all quantitative traits were summarized at the species-level (unless specified) by descriptive statistics in the lower left panel. Associated metadata are displayed in the middle left panel, and traits accompanied by temperature at the time of observation are marked with a thermometer icon. An example of early life stages is shown for the Leopard coral grouper (Plectropomus leopardus), based on unscaled modified illustrations from Masuma *et al*.^133^, with the pelagic juvenile stage represented by a similar-sized^134^ settlement-stage individual from Leis^135^.

To improve accessibility and clarity of dispersal traits, we compiled three datasets with incubation/gestation duration, pelagic juveniles (*i.e*., demersal species remaining pelagic after metamorphosis from larvae to juvenile), and rafting (*i.e*., drifting passively next to objects/animals) behaviors records. We also separated datasets about metamorphosis and settlement age/size (Fig. 1), to clearly isolate traits specific to demersal species (*i.e*., settlement and pelagic juveniles). Additionally, we recorded the duration of metamorphosis (*i.e*., one to hundreds of days) based on the authors’ definitions for each species, though it typically involves changes in pigmentation, the onset of scalation, and/or the fixation of adult meristic traits^48,49^. The age and size for all intermediate early life stages was also retrieved: hatching (size only), first feeding (start of exogenous nutrition), yolk-sac absorption (complete depletion of egg resources), and flexion (upward notochord bending associated with the ossification of fins). Indeed, the switch toward exogenous nutrition is generally associated with the onset of vertical migration^50^ and the main mortality peak^51^, while it is from flexion that larvae usually acquires efficient horizontal swimming abilities^52–55^ associated with great endurance capacities^56^. Although swimming speed changes with age, we restricted our analysis to measurements from individuals at or near the settlement stage, as only a limited number of studies have assessed swimming performance across the entire larval period^57^. We separated forced speeds measured in swimming flumes (critical swimming speed: U_crit_) from routine speeds observed in the wild via scuba diving (*In Situ* Swimming speed: ISS) as they usually provide very different estimates^23,58^, and decided not to record orientation accuracy since it was already recently reviewed^22,25^. Additional dispersal-related traits included in our dataset encompass reproductive mode, which influences whether dispersal occurs during the egg stage^47^, egg characteristics (*e.g*., presence/size of oil globules which partly affect buoyancy^59^), and larval vertical distribution influencing passive drift, as currents are generally weaker at depth. Along with species-level (or higher-level) traits, we digitized individual-level age-at-length/width data (latter referred as “growth rates” since various models are based on it) from rearing experiments to enable temperature-associated growth modeling, addressing the limitation of previous reviews that reported larval growth model parameters without temperature^60,61^. Finally, various traits were further completed by additional measurements carried out during two fieldworks in the Caribbean and the Indian Ocean^25^, as well as numerous unpublished data or non-accessible datasets directly obtained from different authors.

**Fig. 2:**
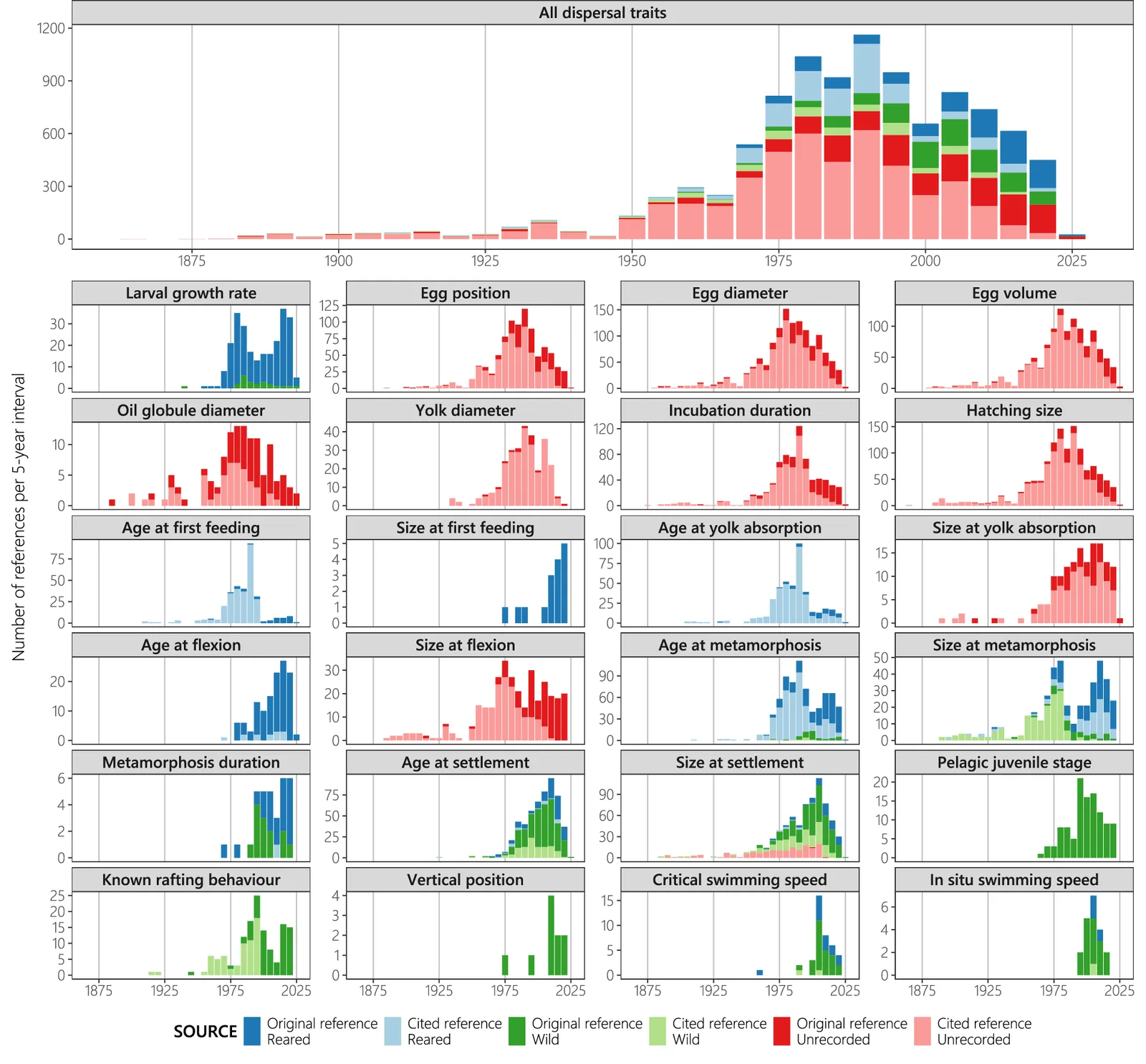
Publication rate per year (5-year time bin) for dispersal traits recorded, type of reference, and type of origin (reared or wild individuals) when recorded. An “original reference” refers to the consulted reference (either the source study or a review) while a “cited reference” corresponds to unchecked references mentioned in reviews (i.e., inaccessible or not necessarily as all metadata were provided). Note that the 2025–2030 interval only contains one year as our review only includes articles before 2026.

Although dispersal trait datasets were not intended to be exhaustive, and reviewing efforts were unequal among variables (*e.g*., first feeding vs. settlement), this initiative to gather relevant data from a large body of literature provides a resource that may largely facilitate future marine fish connectivity studies. Rather than relying on subjective and often unrealistic parameter values^18^, users can now efficiently synthesize results across multiple studies. They can also infer missing data for their focal species using values from related taxa using our datasets, which will ultimately enable more precise multi-specific modeling^17^. Together with the orientation accuracy review of Berenshtein *et al*.^22^, both swimming speed and vertical position datasets should motivate modelers to implement active behaviors as their influence appears of major importance to accurately model dispersal patterns^20,47^. However, they have not been included in the vast majority of dispersal models to date^18^. Moreover, biophysical models for demersal fishes have not yet explicitly considered facultative or mandatory pelagic stage prolongation to our knowledge, despite its likely major impact on large-scale connectivity. This is a question that can now be addressed using our pelagic juvenile and rafting datasets. Unlike most previous reviews that report only species-level means or ranges (*e.g*.^62,63^), our systematic inclusion of full descriptive statistics associated with the development of a robust method to obtain probability density functions (PDFs) from them, should facilitate the integration of trait variability into biophysical models^64–66^. Beyond biophysical modeling, we hope that the availability of these traits will revitalize research in the recently declining field of marine larval biology and ecology (Fig. 2). In particular, reporting temperatures metadata based on each article methodology for almost all dispersal traits (Fig. 1) might help forecast the effects of global warming on larval biology and connectivity^52,67,68^. Finally, our comprehensive review of larval rearing experiments provides fish farmers, public aquariums, hobbyists and environmental managers with a centralized resource on protocols used in successful culture through metamorphosis, which may help efforts to reduce the predominance of wild-caught fish in the aquarium trade^29^.

## Methods

### Bibliographic review

#### Traits search and species filtering

We performed our search of dispersal traits for marine fishes sequentially, starting by processing large sources of information (*e.g*., automated extraction methods, bibliographic reviews) before refining it using additional Google Scholar searches as well as manual sources reanalysis, to make sure the greatest amount of data could be retrieved from each reference. For each reference, we aimed to obtain species-level descriptive statistics of numeric values along with associated temperatures for each independent location and period per species (Fig. 1). We also systematically reported the origin of individuals (or set to “Unknown”), the type of length when relevant (*i.e*., SL = standard length, TL = total length, NL = notochord length, FL = fork length), and the source of information (*i.e*., cited references in reviews if available, standardized original reference short name and URL/DOI; Fig. 2). Most data processing, including table creation, standardization, and validation, was conducted using R version 4.5.0. Qualitative dispersal traits or metadata were standardized in the lowest number of categories possible (except for remarks columns). We systematically obtained valid names from WoRMS (04/2025) using the function *wormsbynames* (package worms^69^) by correcting typos and searching for registered synonyms in WoRMS within the Eschmeyer Catalog of Fishes for names without matches. All non-actinopterygians and freshwater species were systematically detected and removed from each dataset, except for species found simultaneously in freshwater, brackish and marine ecosystems, or brackish and marine ecosystems according to the package rfishbase^70^. We also removed anadromous and potamodromous genera which were defined as containing at least one species registered as such by FishBase (package rfishbase followed by manual curation/completion), since their larval phase does not occur in the marine environment. Pelagic species as adults were excluded from the juvenile and settlement datasets by first removing non-reef species based on the GASPAR dataset^71^, followed by manual verification of habitat information in FishBase for species listed as “bathypelagic”, “pelagic-neritic”, or “pelagic-oceanic” (package rfishbase followed by manual curation/completion), retaining only those whose juveniles occur on the seafloor or in very shallow waters.

#### Semi-automated searches of settlement ages

**Fig. 3:**
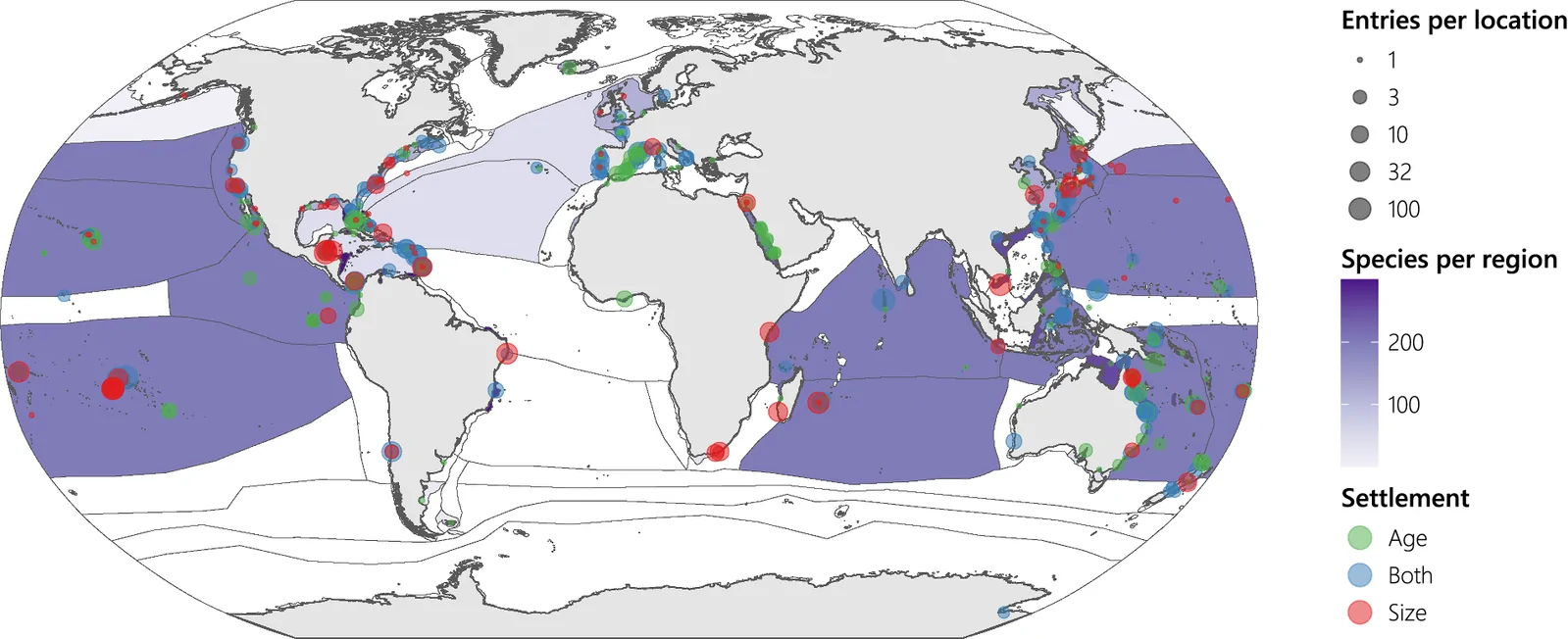
Sampling locations from which wild individuals were retrieved to determine their settlement size or age through otolithometry in references reporting the sampling location. The radius of points is proportional (logarithmic scale) to the number of records (number of individual periods and species) for each GPS point. Marine Ecoregions^136^ and Pelagic Provinces^137^ of the world were colored by the number of species for which the age and/or the size is known.

First, we used the software Perish or Publish 8^72^ to automatically obtain a list of articles about PLD in coral reef fishes (*i.e*., initial scope before shifting it to all marine fishes) using the following Google Scholar query: *“pelagic larval duration” AND ~otolith AND (“fish larvae” OR “larval fish” OR “ichthyoplankton”) AND “coral reef”*. After automated download within Zotero (for open-access/available articles), we used the R package PDE^73^ to convert tables within each PDF in Excel format by detecting them based on the following keywords: *PLD;pelagic larval duration;larval duration;pelagic duration;pelagic phase duration;durée larvaire;durée pélagique;duración pelágica;duración larvaria;duração larval;duração pelágica*. The same procedure was applied to obtain the swimming speed using an adapted Google Scholar query (*((“in situ swimming” AND ~diver) OR (“in-situ swimming” AND ~diver) OR “critical swimming speed”) AND (“fish larvae” OR “larval fish” OR “ichthyoplankton”) AND “coral reef”*), and new keywords for table extraction: *swimming speed;speed;in situ;ISS;in situ swimming;Ucrit;critical speed;Ucrits;cm/s;cm.s;cm s;bl/s;bl s;bl.s*. Each Excel sheet formatted with a custom macro script for easier use (*e.g*., scaling, keyword highlighting), as well as PDFs for which information could not be extracted automatically, were manually analyzed to detect relevant information. Preliminary tables created using this method were carefully verified and complemented with in-text information such as additional metadata (*e.g*., sampling date/gear/locality), by directly searching it in the primary study (Fig. 3). In particular, regions were determined for each named locality following the regionalization of Miller *et al*.^74^, and those designating sufficiently precise areas (<100 km²) were assigned approximate GPS coordinates if they were not already provided. Primary studies analysis also enabled the selection of swimming speeds measured specifically at the settlement stage, and allowed the separation of references related to metamorphosis or settlement age, as well as the division of critical and *in situ* swimming speeds (excluding other types of speed measurements) into distinct tables. The same meticulous approach was used to reanalyze all references from a private dataset listing many “PLD” records until 2014 (including 122 unpublished ones from 9 different authors), which finally accounted for 6% of all settlement-age dataset records (Kulbicki, unpub.). Building on this comprehensive initial literature record, we expanded it through forward and backward snowballing, an approach shown to be highly effective for identifying primary studies^75^, and broadened the scope from coral reef fishes to all marine fishes. It has to be noted that we excluded coarse settlement age estimates derived from the difference between estimated spawning and settlement dates, whether defined empirically or back-calculated using extrapolated larval growth rates from growth models calibrated for adults^76^.

### Creation of other traits datasets

Second, we used size information collected during the development of the metamorphosis/settlement age datasets (*i.e*., both information in the same articles) to create initial metamorphosis/settlement sizes datasets with similar metadata columns. We substantially increased them using a similar snowballing review approach, along with acquiring individual-level size from annotated pictures in books (*e.g*.^77,78^), genetic datasets (*e.g*.^79,80^) or websites (*e.g*.^81^). We also carried out new size measurements based on scaled pictures of settlement-stage fishes collected by light traps in Vietnam^82^, using a Python application named Django Labeller for image segmentation and labeling^83^, along with a custom automatized Python script for linear or non-linear lengths computations (225 images for 86 species). Unpublished settlement sizes (126 records) were also obtained from B. Victor, most of them being visible on his website^84^ or BOLD. Searching for metamorphosis sizes measured in wild individuals, we gathered a large corpus of identification resources reporting sizes at earlier larval stages (*e.g*., hatching, flexion) along with information about eggs (*e.g*., pelagic/benthic spawning, egg size/shape; Fig. 1). Individual-level datasets (*e.g*.^76,85^) were summarized by computing weighted means and standard deviations using the package Hmisc^86^. Each online website (*e.g*.^87–90^) was harvested using custom scripts based on the R package rvest^91^. Similarly, each book in which information was categorized (*e.g*.^26,92^) was converted to a text format (non-selectable PDF converted with this OCR algorithm: https://www.ilovepdf.com/ocr-pdf) for semi-automatic data extraction with custom R scripts per book. We also used an online OCR algorithm named Nanonets (https://app.nanonets.com/#/) specifically trained to extract tables in CSV from PDF files (*e.g*.^37,63,93^) before manually formatting them, and/or manually extracted in-text values from other references (*e.g*.^94,95^). In addition, early life-history traits from FishBase^96^, including egg data not accessible with the rfishbase^70^ package (unified table obtained from a collaboration with the FishBase Team), were also integrated into these new datasets, contributing 24.5% of the egg-related records, 9.1% of incubation durations, 6.2% of hatching sizes, and 2.2% of flexion sizes. No FishBase data on metamorphosis size or metamorphosis/settlement age were included, as equivalent records were already present following thorough dataset comparisons to avoid introducing duplicated values.

#### Dataset completion with aquaculture studies

Third, we completed the metamorphosis and settlement age datasets, as well as earlier age thresholds between larval stages, with information reported in aquaculture studies, also enabling us to enrich size datasets (Fig. 2). Various extensive review tables in the book of Tucker^33^ served as a starting point for a forward snowballing review primarily aiming at building a comprehensive literature record after 2000. Additionally, we also systematically searched for publicly available aquaculture records of all reared marine ornamental fishes based on two comprehensive checklists^28,29^. To do so, we used the following query in both Google and Google Scholar for each species name in these lists: (“Genus species”) AND (metamorph* OR transition OR transform*) AND (rear* OR breed* OR bred* OR mariculture OR aquaculture OR culture). Apart from searching information in primary studies along with useful metadata (*e.g*., sample size, temperature), we also retrieved relevant data from recent reviews (*e.g*.^35,36^) and websites or blog posts (*e.g*.^97,98^). We applied the same methodologies as described above to gather dispersal traits from these varied sources, allowing us to obtain ages at the end of intermediate larval stages that could otherwise be difficult to estimate from wild individuals. While analyzing each aquaculture reference, we set aside those providing size or weight across age for at least four approximately spaced age points, covering at least two-thirds of the larval (pelagic fishes) or pelagic (benthic fishes) phase, according to the previously established metamorphosis/settlement size datasets. Based on this literature record enriched with some published growth rates measured on wild individuals using otoliths, we extracted individual-level measurements from plots using PlotDigitizer (https://plotdigitizer.com/app), from tables using Nanonets, and from images (size measurements) using Django Labeller, as described above. Such a dataset was completed by analyzing accessible references in aquaculture reviews that indicated which one corresponded to successful rearing experiment until metamorphosis/settlement^33,40,99,100^, as well as age-length data from the ontogenetic speed dataset of Fisher *et al*.^38^.

#### Swimming speed, pelagic juvenile and rafting datasets

Fourth, we focused on creating various datasets about behaviors (*i.e*., rafting phase, pelagic juvenile phase and vertical migration; Fig. 1) possibly affecting dispersal along with swimming speed and orientation accuracy (reviewed by Berenshtein *et al*.^22^. We used the comprehensive review of Castro *et al*.^46^ as a starting point for a forward and backward literature review of all references reporting rafting behaviors in larval, juvenile, or adult marine fishes (Fig. 2). We completed such review with an extensive Google Scholar search using the following query for articles published between 2002 and 2024: marine AND fish* AND (raft* OR flotsam OR floatsam OR “drift object” OR “floating object” OR “Fish Aggregating Device” OR “tsunami debris” OR pumice). A non-standardized Google Scholar search was also performed to find references listing long-term associations between fishes and gelatinous zooplankton or large vertebrates in the pelagic ecosystem. Although the latter does not correspond to passive drifting, it could largely extend dispersal potential, which is the reason for the creation of a second dataset listing all juveniles of demersal/benthic fishes^96^ sampled in the pelagic environment. To do so, we performed five consecutive Google Scholar searches using a simple query *(“keyword” AND fish*), for these keywords: *“pelagic juvenile”*, *“pre-settlement juvenile”*, *“presettlement juvenile”*, *“glass eel”* and *“pelagic prejuvenile”*. Records using the latter keyword (28%) were excluded due to its ambiguity, as some authors apply it to pelagic juveniles while others refer to the late post-flexion stage. Along with qualitative variables (*e.g*., flotsam type, stage, keyword) in both pelagic juvenile and rafting datasets, we also recorded the size and/or age (or duration if specified) of sampled individuals for primary studies and reviews reporting it. As the maximum size of pelagic juveniles was generally equivalent to or higher than the settlement size, we included it in the settlement size dataset while specifying in a specific column that these records do not correspond to actual sizes measured at settlement.

#### Vertical distribution dataset

Finally, we assembled a dedicated dataset on larval vertical distribution, by searching Google and Google Scholar with the following query: *(“stratified net” OR “stratified sampling” OR MOCNESS) AND “fish larvae”*. After compiling raw datasets, including unpublished data obtained by contacting authors^101,102^, we only retained studies that sampled the water column at least between 0 and −100 m, using depth intervals of less than 30 m, and covering the entire sampled range. This choice prevented us from considering coarser stratified sampling (*e.g*.^103^), shallow stratified sampling (*e.g*., nearshore sampling^104^), and recordings below 100 m. Indeed, although some datasets extended to 200 m or deeper, they lacked the fine depth resolution needed^105,106^, and those with fine resolution until 150 m were too few to justify the creation of a dataset deeper than 100 m^104,107^. While our focus was on demersal and epipelagic species, their larvae can occasionally be found much deeper than −200 m^108,109^. Nevertheless, the only nearshore study of those cited above reveals that samples identified at the species-level and whose adults are epipelagic or demersal represent 96.1% of the ichthyoplankton community above 100 m, while they make only 36.7% of standardized larval densities between 100–210 m compared to bathyal species^105^. For each species, life stage (pre-flexion, flexion, post-flexion), zone, and diel period (day/night), we computed mean depth by weighting depth-bin midpoints with standardized larval densities (individuals per 100 m³, or per m² when net volume was unavailable), to correct for unequal filtered volume across nets. Unlike other datasets, we retained genus-level records in the rafting dataset, and included subfamily- and family-level entries in the vertical distribution datasets (excluding order-level), to account for the high proportion of unidentified taxa (10% and 41%, respectively; Fig. 1).

### Fieldwork measurements

#### Sampling, processing, and measurements

Samplings were conducted in Goidhoo Atoll (Maldives, 04-05/2024, GPS: 4.839800, 72.963697) and three different locations around Guadeloupe island (Caribbean, 01-04/2023): Vieux-Fort town (GPS: 15.961553, −61.709503), Gosier Island (GPS: 16.196573, −61.492215) and Fajou Island within a marine reserve (GPS: 16.35937, −61.574622). Sampling employed a combination of gears, primarily light traps (CARE, Ecocean) and custom crest nets (1 mm mesh size) deployed overnight, as well as longer-duration custom-made traps (FARMS^110^; SMURFs^111^), and hand nets (0.5 mm mesh size) used while snorkeling, scuba diving, or from boats. We mainly captured settlement-stage fishes which were used both for settlement size and ISS measurements (see detailed protocol in Ruiz *et al*.^25^), but also settled juveniles/adults (mostly using FARMS and SURMFs) from which we also estimated settlement age using the settlement mark on otoliths^112^. After each sampling session, we quickly sorted fishes by morphotype and photographed against both a fully black and fully white background one or two representative individuals per batch, selecting alive individuals if possible, or the least damaged dead fish. We then euthanized these tropical species by quickly cooling them in a freezer and then placing them in a fridge before ice formed, with the approval of the country in which fieldworks were carried out. All fish’s manipulations were performed in compliance with research permits delivered by the Direction de la Mer, Guadeloupe (No. 665/2022 & No. 158/2023), and the Ministry of Fisheries and Ocean Resources in the Maldives (No. NRP2023/63). Dead fishes were then morphologically identified to the lowest taxonomic level possible based on meristic counts and body shape/color using a wide range of identification resources (*e.g*.^32,77,113–115^). In the Maldives, unidentified live fishes were placed in transparent vials with finely pierced caps (water renewal) set at sea for several days in floating keepnets, to ease identification based on settled juvenile color patterns. We made sure the overall process lasted less than two days after death since fishes were ultimately dissected to extract muscle, or the full body without digestive parts and head. These samples were conserved within Longmire buffer^116^ in the Maldives or 96% ethanol in Guadeloupe. Among all stored samples, doubtful morphological identifications or under-represented species in local genetic datasets were selected for DNA sequencing. All species-level identified settlement-stages photographed against a black background better revealing fins outlines were measured using the Django Labeller program (as described above) calibrated with the ruler placed on each frame. Twelve additional settlement size measurements corresponding to 9 new species for this dataset were added based on various samplings in the Caribbean Sea over several years (Victor, unpub.). We also used Django Labeller to measure the 4 rafting species (all juveniles) detected during fieldwork around floating plastic (*Abudefduf vaigiensis*, *Caranx bartholomaei* & *Paramonacanthus pusillus* never recorded before as a rafter) and a dead log (*Kyphosus vaigiensis*).

#### Genetic identification of samples from Guadeloupe

For Guadeloupe samples, tissular DNA extractions were carried out from various dissected parts (*i.e*., whole bodies without digestive tracts to muscles fragments) using the DNeasy Blood & Tissue Kit (Qiagen), which involved a 20 mn digestion step at 56 °C, followed by a custom-made protocol modified to perform a final double-elution step (50 μL each) within a Qiacube (Qiagen) automate, and therefore maximize DNA concentrations later checked with a Qubit 4 (Invitrogen). Fragments of 655 bp from the cytochrome oxidase I (COI) gene were first amplified using the reverse primer FishR1 and a cocktail of forward primers FishF1 and FishF2^117^. PCR reactions (40 μl total volume) contained 20 μl MyTaq PCR Mastermix (Bioline), 16 μl ultrapure water, 0.8 μl BSA (Euromedex), 0.6 μl of each primer (3 μM), and 2 μl DNA template. Amplification was conducted on a PTC-100 (Bio-Rad) thermal cycler with an initial denaturation at 92 °C for 2 min, followed by 35 cycles of 92 °C for 45 s, 52 °C for 45 s, and 72 °C for 1 min, with a final extension at 72 °C for 5 min and a cooling at 4 °C. PCR products were visualized on 1–2% agarose gels, and the most intense bands were selected for Sanger sequencing (Genoscreen). Electropherograms were quality-checked using 4Peaks^118^, and sequences were aligned with MUSCLE in MEGA7^119^, followed by manual verification. Preliminary identifications against NCBI with blastn^120^ allowed us to remove samples contaminated with bacteria (probably rotten before conservation) before creating a BOLD project (https://doi.org/10.5883/DS-GWAFI) for automated identification, BINs attribution and submission of sequences to GenBank. All retained 145 samples were reidentified against a curated reference dataset focused on Caribbean reef fishes (Victor, unpub.) to detect errors/uncertainties within the BOLD dataset, and to benefit from additional private sequences. Meanwhile, a ~675 bp region of the 12S gene was amplified for 57 samples, including 17 not previously sequenced, for a global assignment of the fragment against GenBank. As this region contains the Teleo1 metabarcode^121^, assignments were also performed for this fragment against a curated reference dataset (Bruno *et al*., in prep). Briefly, sequences were prepared following the methodology of Rozanski *et al*.^122^: amplification was performed using the V05F-898/Teleo-R primers^121,123^ and the REDExtract-N-Amp PCR ReadyMix (Sigma), before sending amplicons to Eurofins Genomic for Sanger sequencing. Overall, we could confirm or correct morphological identifications for 75 species out of 92 (most other morphological IDs were sure) used in settlement size/age and rafting datasets, including 11 solely using the 12S gene, as well as 42 using both genes. Scaled pictures on a white background that can be used for identification, morphometric or illustration purposes were added to the BOLD project for 113 individuals out of 157 (66 species).

#### Genetic identification of samples from the Maldives

For Maldives samples, DNA extractions were performed with the Promega Maxwell HT 96 gDNA Blood Isolation System kit. All extractions were automated with a KingFisher Apex Sytem, for a final elution volume of 160 μL for 56 samples, and 80 μL for 367 samples (custom-made protocol). DNA concentrations were checked with a Spark Plate Reader (Tecan), enabling selecting the two samples per species with the greatest DNA concentration for amplification of both COI and 12S. PCR reactions targeting a 650 bp fragment were set up in 10 μl volumes using 0.5 μl (10 μM) of a cocktail^124^ of forward primers (VF2_t1 + FishF2_t1), and reverse primers (FR1d_t1 + FishR2_t1), along with 5 μl Taq Sigma (ReadyMix PCR REDExtract-N-Amp), 3.5 μl ultrapure water, and 1 μl DNA template. Thermal cycling was carried out on a Mastercycler X50 (Eppendorf) with an initial 2 min denaturation at 94 °C, followed by 35 cycles of 94 °C for 30 s, 52 °C for 40 s, and 72 °C for 1 min, ending with a 10 min final extension at 72 °C, followed by cooling at 4 °C. Amplification success was checked on 1% agarose gels, and the most distinct bands at 650 bp were selected for downstream Sanger sequencing. Unlike Guadeloupe samples, the complete 12S was amplified following the protocol of Volanandiana *et al*.^125^: using the XRMUPheF1 (or Phe12S-F in case of mis-amplification, a newly designed primer for this study: 5’-ATCAAAGCRTRACACTGAAG-3’) and Teleo-R^121^ primers to obtain the full gene with a single PCR. To obtain a clean chromatogram for the 3’ end of the 12S gene, Eurofins sequenced in the forward direction both the full gene (XRMUPheF1 / Teleo-R) and the MiFish-U-F / Teleo-R^126^ fragment. Chromatogram corrections were semi-automated with the R packages sangerseqR^127^ and DECIPHER, in combination with the website Pearl (https://www.gear-genomics.com/pearl/^128^), before manual verification with 4Peaks and MEGA7, preliminary assignment on NCBI, and final assignment on BOLD as for Guadeloupe samples. Overall, we could confirm or correct morphological identifications for 152 species out of 177, generating new COI sequences for 17 species previously not recorded in BOLD, and new complete 12S sequences for 71 species previously not in NCBI. Every sequence and associated pictures will be public soon as for Guadeloupe samples.

#### Settlement age measurement

**Fig. 4:**
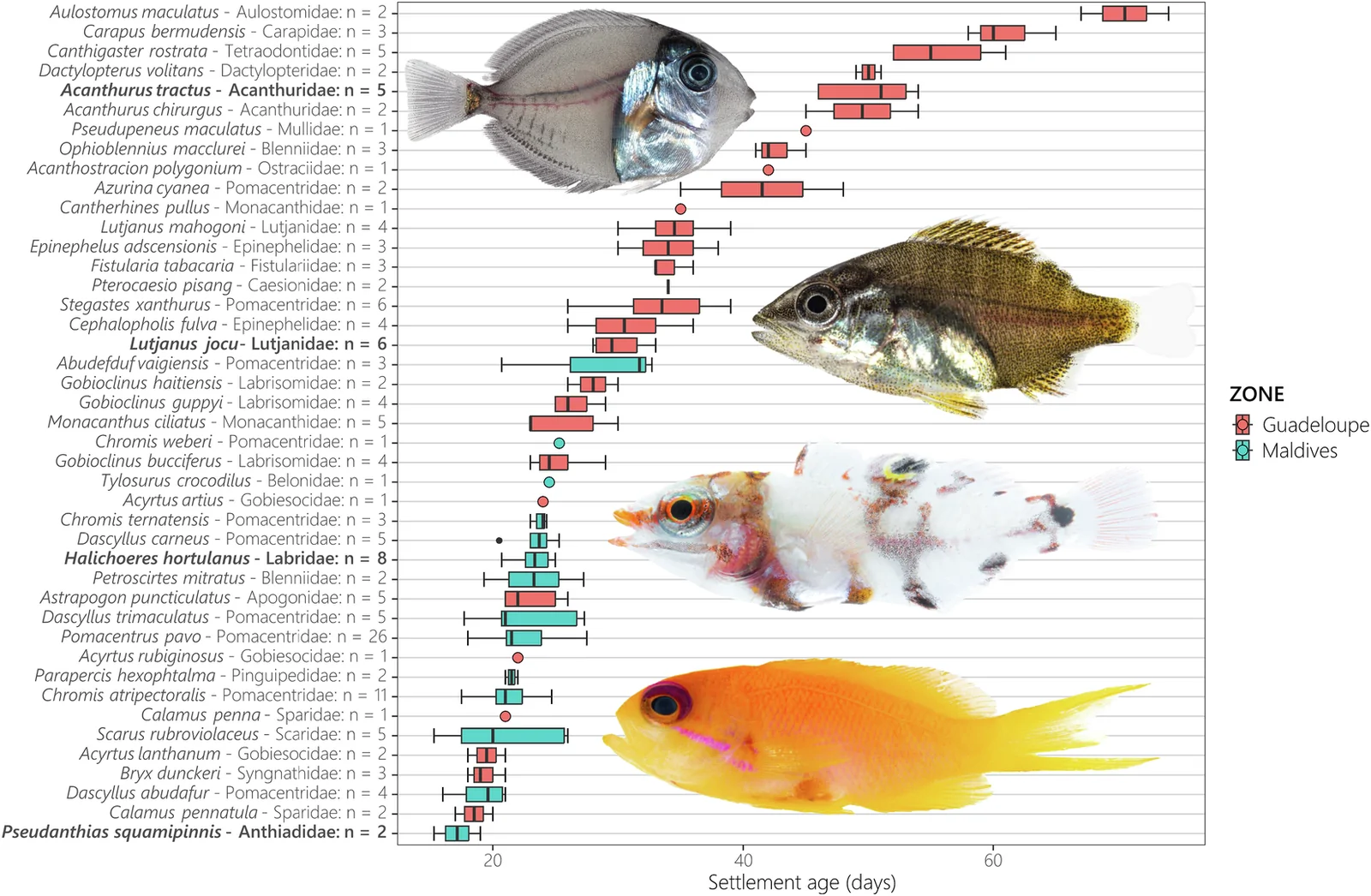
Newly measured settlement ages from otoliths of fishes captured during fieldworks in Goidhoo Atoll (Maldives) or Guadeloupe Island (Lesser Antilles). Examples of pictures systematically taken against a black and a white background are shown next to the corresponding species name highlighted in bold. These pictures show two settlement-stage individuals collected in Guadeloupe, for which settlement age was estimated from total otolith age, and two settled juveniles from the Maldives, for which settlement age was determined by counting daily increments on otoliths until the settlement mark.

**Fig. 5:**
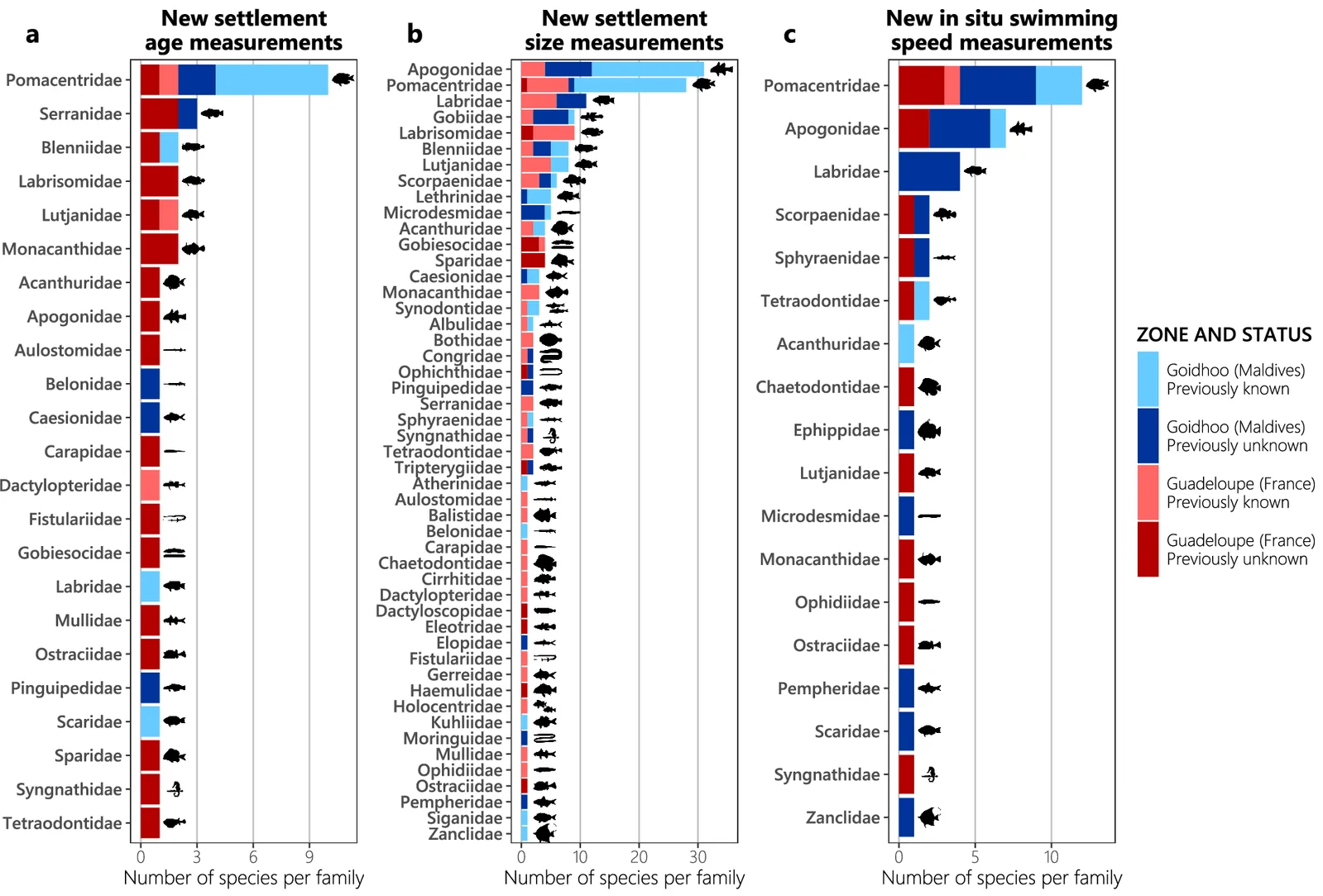
New settlement age (**a**), settlement size (**b**) and *in situ* swimming speeds (**c**) recorded during fieldworks in Goidhoo Atoll (blue) or Guadeloupe Island (red). Newly recorded species compared to datasets established from the bibliographic review are presented by dark colors, while species for which at least one measurement was already reported are indicated by light colors in the bar charts. Details on the protocol used to measure new *in situ* swimming speeds are available in Ruiz *et al*.^25^.

Moreover, based on the settlement age dataset established from the bibliography and the result of fieldwork samplings, we selected 26 species for which settlement parameters have not been estimated. Needs for other ongoing projects also led us to measure new settlement ages for 12 additional species with at least one known previous published estimate according to our dataset (Figs. 4 & 5). Otolith extraction was performed within 96% ethanol under a binocular with polarized light illumination for an easier visualization of otoliths, which were generally extracted by the gills, but also for some specific morphologies with a frontal section of fishes’ head^129^. Whenever possible, all three otolith pairs were extracted, photographed, and stored, but we always used the right sagitta for age estimation, while the left sagitta served as a replacement if the right one was damaged/lost or the preparation had failed. After drying vials containing the selected sagitta in an oven (Heraeus) at 35 °C, it was gently placed on an epoxy resin base (Escil Araldite 2020) which had previously solidified in a silicone mold. Otoliths were carefully positioned under a binocular with their internal face downward, aligned at two-thirds the length of the rectangular mold and centered laterally considering the core, with the rostrum oriented lengthwise towards the nearest edge. A drop of epoxy resin just prepared was slowly poured onto the otolith to avoid introducing air bubbles, assuring that its initial position was maintained. After waiting 24 hours for resin polymerization in an oven (35 °C), an otolith transverse section of 400–800 µm in width was prepared using a low speed precision saw (Buehler IsoMet 1000), ensuring that the core is present within it. Otolith sections were then grinded and polished by hand. Controlling with a microscope (×200 to ×400 magnifications), the section thickness was progressively reduced on one side until the core level, with different wet abrasive papers (800, 1200, and 2400 μm), before polishing it using 1 µm alumina powder with water^129^. We stopped this process when the core plan was about 20 μm below the surface of the otolith section (estimated using the microscope’s micrometer screw), which is generally noticeable thanks to radiating protein fields and converging daily increments around it, as well as the narrowing/deepening of the sulcus groove on the proximal edge. Otolith sections were then placed for 5 min in an ultrasonic bath (Elma, Elmasonic ONE) to remove debris, and then dried at 35 °C in an oven. The polished side was glued on a small glass slide with a drop of melted thermoplastic Crystal Bond^TM^ quickly poured to avoid introducing air bubbles, and then removed fast enough from the hot plate (140 °C) to prevent the epoxy resin in which the otolith is embedded from melting too^130^. The upward-facing side of the otolith is sanded, cleaned, and dried similarly to the first one before the small glass slide is glued to a microscope slide with Crystal Bond^TM^. Under high microscope magnification (×400 to ×600) according to the otolith size, daily increments were counted on otolith sections by slightly focusing above the core plane to produce a blurred view of the otolith, which makes it easier to distinguish daily from sub-daily increments and to avoid duplicate readings caused by the tilt of increments within the slice depth. The otolith reading path was located within a 45° angle around the axis between the core and the ventral edge, since it is the maximum growth axis on which the increments are best differentiated, even if we sometimes had to count toward the dorsal edge (*e.g*., due to dark protein fields masking increments). Three different readers (except for one reading session in which there were only two) counted increments blindly (*i.e*., not knowing the species/stage/length) during separate sessions, before recounting together otoliths for which the coefficient of variation among age estimates exceeded 10%, to obtain consensual means or discard ambiguous otoliths^129^. If otoliths from post-settlement individuals were used (*e.g*., FARMS, SMURFs, or hand nets samplings in the Maldives), we estimated the age at settlement by counting increments until the settlement mark, which is generally noticeable thanks to marked changes in increment width (growth rate increase/decrease), growth axis change and/or general otolith coloration due to differences in protein content^112,130,131^.

## Data Records

The dataset was attributed to a specific DOI by depositing it on Figshare repository^132^ and the detailed description of its structure is provided on the associated GitHub repository: https://github.com/ruizeliot/fish_larvae_traits_db. Briefly, a total of 7 categorical variables and 28 numerical variables constitute the core of our dispersal traits dataset which were placed in 17 different tables (Fig. 1; see the Github page for specific descriptions) for each main category of trait (*e.g*., the egg table gathers 5 dispersal traits). We adopted a standardized way to provide all statistical descriptors of numeric variables in separate columns identified by a specific suffix in their names: mean (“_MEAN”), min (“_MIN”), max (“_MAX”), variability (“_CONF”), mean type (“_MEAN_TYPE”), range type (“_RANGE_TYPE”) and variability type (“_CONF_TYPE”). These columns were completed by 57 types of metadata considered important for the interpretation of a given dispersal trait (*e.g*., origin, temperature, sample size, variable type, definitions). Metadata columns ranged between 2 (rafting) and 17 (settlement size) depending on the dataset considered, with an average of 8 metadata per dataset. The mean percentage of NA in metadata columns varied depending on the dataset considered, but ranged between 5.5% and 83.3% with an average of 50.2%. In addition, all datasets are completed by 7 taxonomic columns with the classification (up to the order level), AphiaID, and authority for each valid WoRMS name, associated with the original name in the reference. Three reference columns are included: the REFERENCE column provides the abbreviated source name along with the journal name or reference type, the EXT_REF column lists any cited references within reviews, and the LINK column includes the main reference link, with a preference for DOIs over URLs.

## Data Overview

**Fig. 6:**
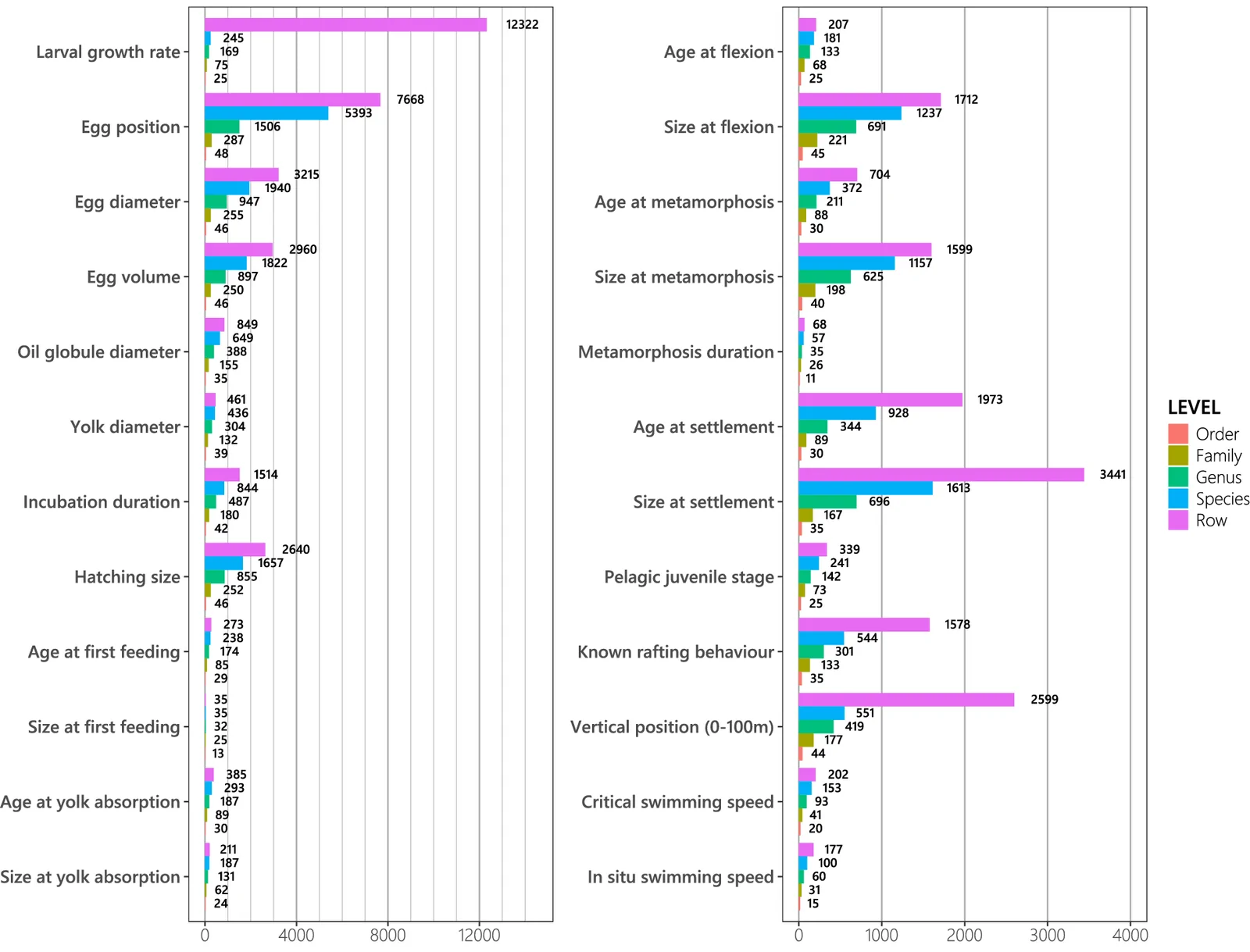
Number of records per dispersal traits and taxonomic levels. Records at the row level correspond to individual-level measurements in the case of the larval growth rate dataset, or to information summarized for each period and location per species within a reference. The exact number of records per case is presented next to the horizontal bars.

Grouped together dispersal traits datasets contain 47,019 records (of which 12,322 are individual-level age/length records) for 6,841 marine fish species, divided into 1,906 genera, 342 families, and 53 orders. Even though it represents only 37% of all marine fishes recorded by FishBase^96^, our dataset covers a large panel of marine fish genera (60%), families (75%), and orders (73%) which should allow users to obtain most needed data by checking values for close species if not available at species-level. However, the taxonomic coverage is very uneven between traits (Fig. 6), with some only measurable on reared fishes (*e.g*., first feeding) being constrained by the small number of marine fish species for which larvae have been reared to the metamorphosis/settlement^28,29^. All data originates from the analysis of more than 5,000 references from which 3,201 references were retained for the dataset, comprising 1,339 primary studies, and 145 references previously reviewing 2,030 studies (Fig. 2). As most reviews focus on wild individuals (*e.g*., field guides, online datasets), the majority of references with reported origin come from rearing studies (61%; Fig. 2), which represent a substantial portion of primary studies. However, improved information detection by combining multiple reviews does not fully explain the drop in publication rates over the past 20–50 years depending on traits since it is observed both for reared and wild-individual-based literature (Fig. 2). Settlement-stage sampling (Fig. 3) is scarce in boreal/polar regions (1% of records) and shows contrasting regional patterns between age-based studies –dominated by the Central Indo-Pacific (23.5%) and Western Atlantic (21.1%)– and size-based records, which are led by the Western Atlantic (27.5%) and Central Pacific (25.1%), with the Central Indo-Pacific ranked only fourth (15.2%). In addition, fieldwork conducted in the Atlantic and Indian Ocean (309 records), as well as unpublished settlement ages (122 records; all obtained from M. Kulbicki) and sizes (126 records; all obtained from B. Victor) allowed adding 128 previously unrecorded species in four key dispersal trait datasets (Fig. 5). In particular, new fieldwork measurements include 38 species (23 families; Fig. 4) for settlement age –68% of which were newly represented– 183 species for settlement size (50 families; 31% new), 42 species for *in situ* swimming speed (17 families; 86% new), and 4 species for rafting (4 families; 25% new). Finally, unpublished vertical position datasets^101,102^ directly obtained from authors enriched this dataset with 907 records covering 106 families (14 previously unrecorded).

## Technical Validation

**Fig. 7:**
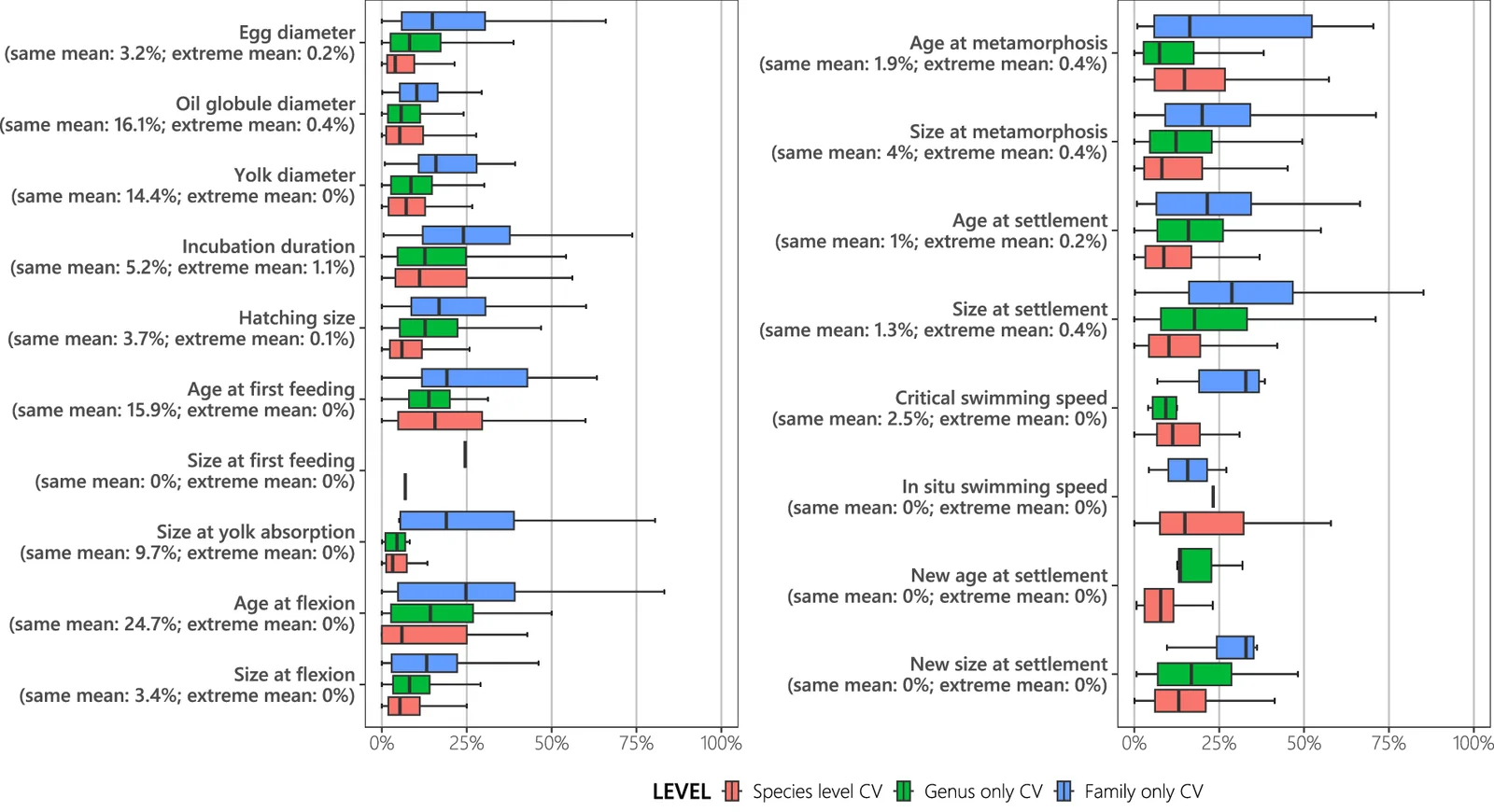
Comparison (coefficient of variation) of each average (mean of midrange) to the species-level mean per species-level numeric traits, or if unavailable to the genus-level or family-level means. The number of equal means (CV = 0%) is indicated on the y-axis names, along with extreme averages (CV > 100%) compared the global mean at the considered taxonomic level. Each record falling in one of those cases was intentionally kept after manually reviewing dataset to remove/correct wrong or duplicated values.

Each dataset comprising both newly acquired data and bibliographic records was carefully reviewed manually. First, we verified within the primary studies all 20 maximum values of each statistical descriptor (mean, min, max, variance) to check for outliers due to conversion problems or decimal typos, and conversely for all 20 minimum values. Secondly, we computed the coefficient of variation (CV) between every numerical record (mean or midrange if only the range was provided), and every species-level, genus-level, and family-level average considering all records in our dataset (Fig. 7). The identification of aberrant records (CV > 100%) compared to averages from all close taxa allowed us to obtain CV for most records, and therefore systematically check records associated with a CV exceeding 100%. This approach also allowed us to identify equal means/midranges for the same species (CV = 0%) which were also manually checked and removed if duplicated (*e.g*., review articles citing the same reference). After this step, mean coefficients of variations per trait were generally lower than 30%, and there was a low amount of similar or extreme records which corresponded in each case to measured values (Fig. 7). Third, each categorical variable was also checked to reduce the number of categories, remove typos, and resolve conflicts if a single species was assigned to multiple categories depending on references. Finally, we verified if the main reviews (*e.g*.,^87^) cited in our dataset comprised all species mentioned in reviewed books and removed additional ones (*i.e*., genus-level means assigned to all congeners within the focal region), while also correcting for any mistakes or adding unreported variables in these reviews.

## Data Availability

The dataset was deposited on a FigShare repository^132^. A comprehensive descriptive of each tables and each column is provided on the associated GitHub page: https://github.com/ruizeliot/fish_larvae_traits_db.
